# Development and validation of a nomogram for predicting overall survival in patients with primary central nervous system germ cell tumors

**DOI:** 10.3389/fimmu.2025.1630061

**Published:** 2025-08-20

**Authors:** Dunchen Yao, Baokui Ye, Hongli Zhang, Long Pan, Dongjie Yao, Xu Li, Chengcheng Guo

**Affiliations:** ^1^ Department of Oncology, The Second People’s Hospital of Guiyang, Guiyang, China; ^2^ Department of Intensive Care Unit, Sun Yat-sen University Cancer Center, State Key Laboratory of Oncology in South China, Guangdong Provincial Clinical Research Center for Cancer, Sun Yat-sen University, Guangzhou, China; ^3^ Department of Oncology, Guizhou Provincial People’s Hospital, Guiyang, China; ^4^ Cordeliers Research Center, Sorbonne Université, Inserm, Université Paris Cité, Paris, France; ^5^ Department of Neurology, Zhenyuan County Hospital, Zhenyuan, China; ^6^ Department of Neurosurgery, Sun Yat-sen University Cancer Center, State Key Laboratory of Oncology in South China, Guangdong Provincial Clinical Research Center for Cancer, Sun Yat-sen University, Guangzhou, China

**Keywords:** primary central nervous system (CNS) germ cell tumors (GCTs), overall survival (OS), nomogram, risk grouping system, Surveillance, Epidemiology, and End Results (SEER)

## Abstract

**Background:**

Primary central nervous system (CNS) germ cell tumors (GCTs) are common neoplasms in the CNS of pediatric and adolescent patients. This study aimed to identify prognostic factors associated with CNS GCTs and establish an effective nomogram for predicting overall survival (OS) in patients with CNS GCTs.

**Methods:**

The development cohort including 1166 CNS GCTs patients was selected from Surveillance, Epidemiology, and End Results (SEER) program between 2000 and 2021. An additional 165 CNS GCTs patients treated at the Sun Yat-sen University Cancer Center (SYSUCC) between 1997 and 2019 were included as validation cohort.

**Results:**

The nomogram incorporated the variables screened by multivariate Cox regression analysis, which included age, sex, histopathology, dissemination, tumor size, radiotherapy, and chemotherapy. The model demonstrated good discriminative performance, with C-index of 0.773 (95% CI, 0.734 - 0.812) and 0.712 (95% CI, 0.599- 0.825) in the development and validation cohorts, respectively. Calibration curves and area under the time-dependent receiver operating characteristic curve (time-dependent AUC) verified the superiority of our nomogram for clinical usefulness. Decision curve analysis (DCA) further illustrated the potential clinical value of the nomogram for treatment decision-making. Additionally, we established a comprehensive risk grouping system that effectively categorized patients into distinct prognostic groups based on their predicted outcomes.

**Conclusion:**

A precise prognostic nomogram was developed for patients with CNS GCTs, utilizing seven independent prognostic factors. It demonstrated satisfactory performance and clinical usability, aiding clinicians in accurately estimating prognosis and guiding the treatment and long-term management of patients with CNS GCTs.

## 1 Introduction

Primary central nervous system (CNS) germ cell tumors (GCTs) are prevalent neoplasms in the pediatric and adolescent central nervous system, representing about 3% of malignant brain tumors in Western countries (1, 2). In contrast, in Asia, particularly in countries such as South Korea and Japan, the incidence ranges from 9% to 15% (3, 4). CNS GCTs are categorized as germinomas or non-germinomatous germ cell tumors (NGGCTs) according to their specific pathological characteristics. The NGGCTs category encompasses teratoma, embryonal carcinoma, endodermal sinus tumor, choriocarcinoma, mixed germ cell tumor, as well as other tumor varieties. Currently, radiotherapy is widely acknowledged for its effectiveness in treating CNS GCTs. With the exception of teratomas, the primary treatment for CNS GCTs involves a combination of chemotherapy along with either whole-brain irradiation (WBI) or craniospinal irradiation (CSI) (5). The rarity of CNS GCTs poses challenges in conducting extensive prospective and retrospective studies, leading to ongoing debates on distinct prognostic factors, such as surgery, tumor dissemination, and sex, and the lack of established models for accurate long-term survival predictions in CNS GCTs. Hence, conducting further research on prognostic factors of CNS GCTs and developing a reliable prognostic model become imperative in order to predict long-term survival outcomes and facilitate individualized treatment and clinical decision-making.

The nomogram was first introduced by Kattan et al. from Memorial Sloan-Kettering Cancer Center in 1998 for predicting the recurrence rate of prostate cancer post-radical resection (6). In contrast to the conventional staging system, the nomogram can incorporate a wider range of prognostic factors to enable personalized predictions. It offers improved predictive accuracy, discrimination, and user-friendliness, making it a more beneficial and convenient tool for predicting survival and stratifying risks. Presently, the nomogram is increasingly employed in predicting the prognosis of different types of tumors (7–13).

In this study, we aimed to identify prognostic factors for CNS GCTs and construct a prognostic nomogram for predicting treatment outcomes based on the Surveillance, Epidemiology, and End Result (SEER) database. Additionally, the nomogram was validated using Sun Yat-sen University Cancer Center (SYSUCC) database.

## 2 Materials and methods

### 2.1 Patient and study variable

The data of CNS GCTs patients from the SEER database (https://seer.cancer.gov/data-software/) were divided into the development cohort. The data were extracted using the following specifications: “Incidence -SEER Research Data,17 Registries, Nov 2023 Sub (2000-2021). The inclusion criteria for patient selection were: (1) histologically proven diagnosis of CNS GCTs; (2) ICD-O-3 codes for histopathology from the 3rd edition International Classification of Diseases for Oncology (ICD-O-3) were utilized to identify cases of CNS GCTs, with the specific codes as follows: 9060/3: Dysgerminomas, 9061/3: Seminoma, NOS, 9062/3: Seminoma, anaplastic, 9064/3: Germinomas, 9065/3: Germ cell tumor, nonseminomatous, 9070/3: Embryonal carcinoma, NOS, 9071/3: Yolk sac tumor, 9080/3: Teratoma, malignant, NOS, 9081/3: Teratocarcinoma, 9082/3: Malignant teratoma, undifferentiated, 9084/3: Teratoma with malignant transformation, 9085/3: Mixed germ cell tumor; (3) Among these codes, 9060/3, 9061/3, 9062/3, and 9064/3 were classified as germinomas, whereas 9065/3, 9070/3, 9071/3, 9080/3, 9081/3, 9082/3, 9084/3, and 9085/3 were classified as NGGCTs; (4) documented survival status and survival time.

To validate the nomogram, we retrieved data from the SYSUCC database for patients diagnosed with CNS GCTs between January 1997 and January 2019, who were assigned to the validation cohort. The inclusion criteria for case selection were: (1) histologically or clinically confirmed diagnosis of CNS GCTs; (2) availability of detailed clinicopathological profiles, complete treatment records, and comprehensive long-term follow-up information. All histopathological diagnoses in this study were verified by neuropathologists at Sun Yat-sen University Cancer Center. The clinical diagnostic criteria utilized in this study are as follows: (1) Typical imaging features along with serum/cerebrospinal fluid levels of AFP (alpha-fetoprotein) ≤ 25 ng/mL and/or HCG(human chorionic gonadotropin) ≤ 50 IU/L indicate germinomas; (2) Typical imaging features along with serum/cerebrospinal fluid levels of AFP > 25 ng/mL and/or HCG > 50 IU/L indicate NGGCTs (14). The tumor markers in blood serum of clinically diagnosed patients in SYSUCC cohort are presented in **Supplementary Table 1**. The primary endpoint of this study was overall survival (OS).

### 2.2 Definition of variables

We utilized the X-tile software to establish the optimal cutoff values for age and tumor size. Age was categorized into two groups: ≤ 21 years and > 21 years, and tumor size was divided into three categories based on the tumor’s largest diameter: ≤ 3.4 cm, > 3.4 cm, and/or unknown. Moreover, the presence of tumor dissemination was identified by either distant metastasis visible in radiographic images or the detection of tumor cells in the cerebrospinal fluid (CSF) at the time of initial diagnosis. In terms of tumor location, the pineal gland and sellar region were merged into a single variable labeled “pineal gland/sellar region,” while all other intracranial sites were grouped as “other sites”.

### 2.3 Statistical analysis

We conducted univariate and multivariate Cox regression analysis in the development cohort to identify independent prognostic factors. Factors with a significance level of p<0.05 in the univariate analysis were incorporated into the multivariate regression model for additional investigation. Then, factors demonstrating significance with p<0.05 in the multivariate analysis were chosen for inclusion in the nomogram development. Based on the contribution degree of each factor for the outcome in the model, the nomogram can express the relationship between various variables in the model according to drawing a line segment with certain proportion on the same plane, which transformed the complex regression equation into graphic visualization, and made the results of the prediction model more reifiable and convenient for clinician.

The predictive performance of the nomogram was assessed using multiple validation metrics, including C-index, calibration curves and area under the time-dependent receiver operating characteristic curve (time-dependent AUC). Following established methodological standards, C-index and AUC values exceeding 0.7 were considered indicative of satisfactory predictive accuracy (15). Furthermore, we implemented decision curve analysis (DCA) to evaluate the clinical utility of the nomogram by quantifying net benefits across a range of threshold probabilities for clinical decision-making.

This study utilized a risk grouping system to evaluate the prognosis of patients with CNS GCTs based on the nomogram. The risk grouping system classified patients from the SEER cohort into four prognostic groups based on their total point scores using the X-tile software. Furthermore, Kaplan-Meier curves were employed to compare the prognosis of patients across the four nomogroups.

Statistical analyses were conducted using SPSS 24.0, incorporating methods such as the chi-square test, Kaplan–Meier method, log-rank test, univariate and multivariate analyses. Significance was determined at a two-tailed P-value of <0.05. R Studio 4.4.1 was utilized for designing and validating the nomogram. The chi-square test was used to assess the correlation between the development and validation cohorts. Survivorship curves were created using GraphPad Prism 7 and compared through the Kaplan–Meier method and log-rank test. The impact of the treatment regimen on CNS GCTs was examined using a Cox proportional hazards model. This study was a retrospective analysis approved by the Institutional Review Board of SYSUCC.

## 3 Results

### 3.1 Patient characteristics

**Figure 1:**
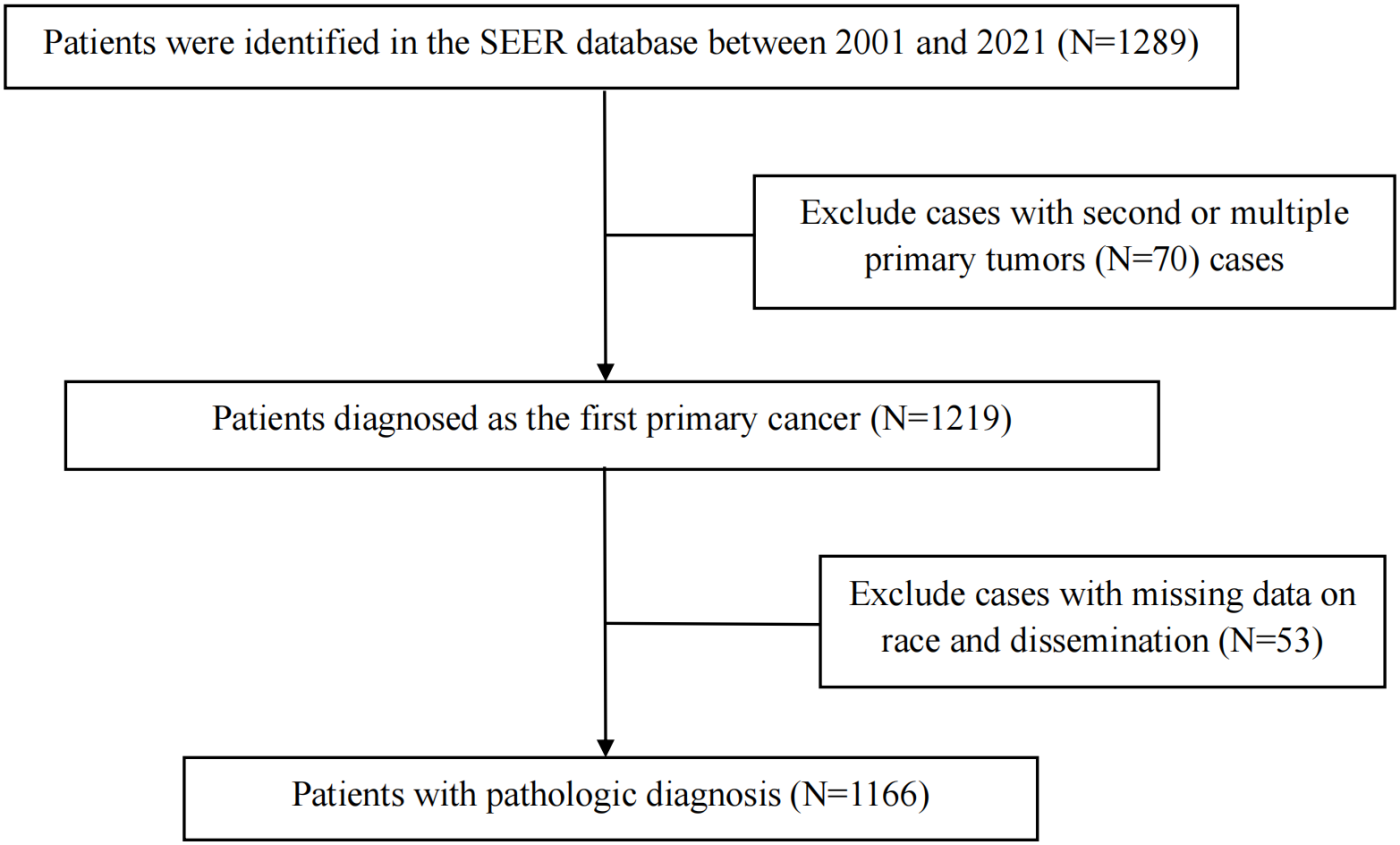
Screening for CNS GCTs patients in the SEER database.

The clinical data of 1289 CNS GCTs patients were extracted from the SEER database from 2001 to 2021. A total of 1166 eligible patients who met the eligibility criteria for CNS GCTs were included (**Figure 1**). Detailed patient demographics and clinicopathological information can be found in **Table 1**. Among these, 911 were diagnosed with germinomas, and 255 with NGGCTs. The 5-year OS rate among patients with germinomas was 93.0%, while for those with NGGCTs, it was 73.3% (**Supplementary Figure 1**). Treatment modalities varied, as shown in **Table 2**, with 28.7% receiving chemoradiotherapy, 10.7% receiving radiotherapy alone, and 6.3% receiving chemotherapy alone, resulting in 5-year OS rates of 94.9%, 91.2%, and 86.3%, respectively (**Supplementary Figure 2**). Tumor dissemination occurred in 90 patients (7.7%), comprising 63 germinoma cases and 27 NGGCTs cases, with corresponding 5-year OS rates of 90.0% and 44.4% (**Supplementary Figure 3**). Within the SEER cohort, the median OS was 109 months (ranging from 0 to 263), with 5- and 10-year OS rates of 88.7% and 87.1%, respectively. The Cox proportional hazards model analysis indicated no significant difference in survival outcomes between germinoma patients treated with chemotherapy versus radiotherapy (HR = 0.586, p=0.286) (**Table 3**). For NGGCTs patients, a more favorable prognosis was observed in those undergoing a combination of surgery and chemoradiotherapy compared to other treatment combinations. However, no significant difference was noted when compared to patients receiving chemoradiotherapy (HR = 1.224, p = 0.609) (**Table 3**).

**Table 1:** The demographic and clinicopathological variables of development and validation cohort.

Variables	Development cohort (n=1166)		Validation cohort (n=165)		P
	No. of patients	No. (%)	No. of patients	No. (%)	
Sex					0.109
Male	910	78.0	119	72.1	
Female	256	22.0	46	27.9	
Age					0.021
Median (years)	16.6		16.6		
<=21	911	78.1	115	69.7	
>21	255	21.9	50	30.3	
Tumor Size (cm)					<0.001
<=3.4	639	54.8	37	22.3	
>3.4	298	25.5	70	42.8	
Unknown	229	19.7	58	34.9	
Histopathology					0.104
Germinoma	911	78.1	119	71.3	
NGGCT	255	21.9	46	27.5	
Tumor dissemination					<0.001
No evidence	1076	92.3	136	82.4	
Yes	90	7.7	29	17.6	
Tumor location					<0.001
Pineal gland/sellar region	666	57.1	134	81.2	
Other sites	500	42.9	31	18.8	
Radiotherapy					<0.001
Yes	913	78.3	165	100.0	
No evidence	253	21.7	0	0	
Chemotherapy					<0.001
Yes	801	68.6	165	100.0	
No evidence	365	31.4	0	0	
Surgery					<0.001
Yes	583	50.0	52	31.5	
No	583	50.0	113	68.5	
Treatment					<0.001
RT alone group	125	10.7	0	0	
CT alone group	73	6.3	0	0	
Surgery alone group	63	5.4	0	0	
RT plus CT	335	28.7	95	57.6	
RT plus surgery	127	10.9	0	0	
CT plus surgery	67	5.7	0	0	
RT plus CT and surgery	326	28.0	70	42.4	
No treatment	50	4.3	0	0	
Diagnosis					<0.001
Clinical diagnosis	0	0	66	40.0	
Pathologic diagnosis	1166	100	99	60.0	

p < 0.05 was considered significant.

NGGCT, non-germinomastous germ cell tumor; RT, Radiotherapy; CT, Chemotherapy.

**Table 2:** Treatment regimen for CNS GCTs patients in the development cohort.

Treatment	Germinomas (n=911)		NGGCTs (n=255)	
	No.	%	No.	%
RT alone group	124	13.6	1	0.3
CT alone group	57	6.2	16	6.3
Surgery alone group	19	2.1	44	17.3
RT plus CT	295	32.4	40	15.7
RT plus surgery	119	13.1	8	3.1
CT plus surgery	26	2.9	41	16.1
RT plus CT and surgery	239	26.2	87	34.1
No treatment	32	3.5	18	7.1

NGGCT, non-germinomastous germ cell tumor; RT, Radiotherapy; CT, Chemotherapy.

**Table 3:** Cox hazard proportional model for CNS GCTs.

Treatment	Germinomas		NGGCTs	
	HR (95% CI)	P	HR (95% CI)	P
RT alone group	Reference		0 (0.-3.089e+229)	0.971
CT alone group	0.586 (0.220-1.564)	0.286	2.736 (1.188 -6.301)	0.018
Surgery alone group	5.058 (2.294-11.148)	<0.001	2.180 (1.123-4.232)	0.021
RT plus CT	0.579 (0.323-1.037)	0.066	1.224 (0.564-2.652)	0.609
RT plus surgery	0.474 (0.216-1.041)	0.063	6.050 (2.390-15.316)	<0.001
CT plus surgery	0.559 (0.130-2.393)	0.433	2.309 (1.176-4.534)	0.015
RT plus CT and surgery	0.354 (0.173-0.724)	0.004	Reference	

NGGCT, non-germinomastous germ cell tumor; RT, Radiotherapy; CT, Chemotherapy; HR, Hazard ratio.

A validation cohort comprising 165 patients with CNS GCTs from SYSUCC was established, consisting of 119 germinomas and 46 NGGCTs. The demographic and clinicopathological data of the patients are detailed in **Table 1**. Among the diagnostic approaches, 99 patients (60.0%) received a pathological diagnosis, while 66 patients (40.0%) were diagnosed clinically. Patients with clinically diagnosed germinomas and NGGCTs have 5-year survival rates of 92.8% and 77.7%, whereas patients with pathologically diagnosed germinomas and NGGCTs have respective 5-year survival rates of 93.6% and 72.8%. As of January 1, 2019, the SYSUCC cohort had a median follow-up time of 70.7 months, during which 21 patients (12.7%) had passed away. The median OS in the SYSUCC cohort was 68 months (ranging from 6 to 197), with 5- and 10-year survival rates standing at 89.7% and 87.8%, respectively.

### 3.2 Kaplan-Meier survival analysis of germinomas and NGGCTs in the development cohort

**Figure 2:**
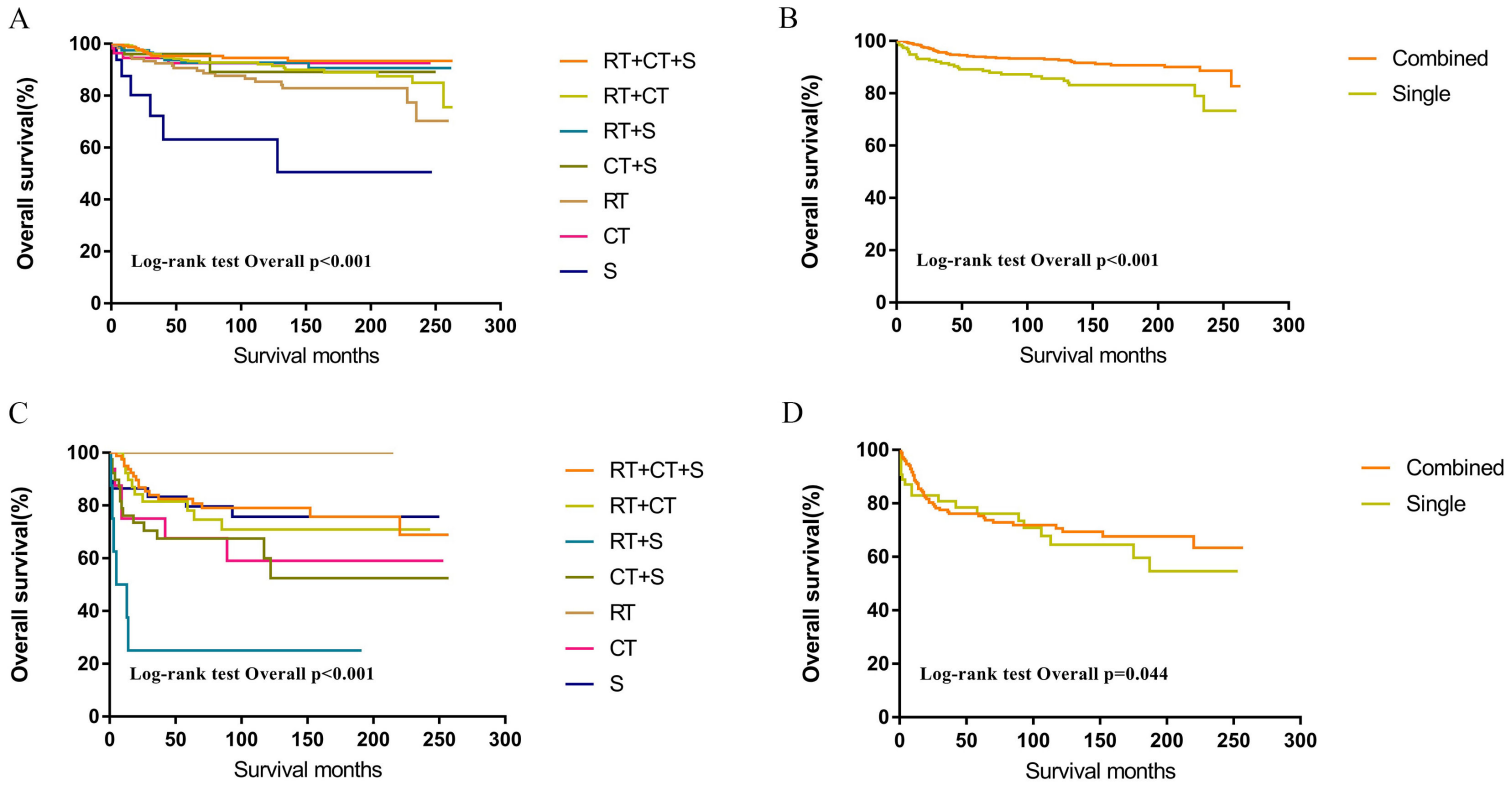
Survival curves of overall survival in germinomas **(A, B)** and NGGCTs **(C, D)** according to treatment regimen. RT, Radiotherapy; CT, Chemotherapy; S, Surgery; Single = RT or CT or S; Combined = RT+CT or RT+S or CT+S or RT+CT+S.

**Figure 3:**
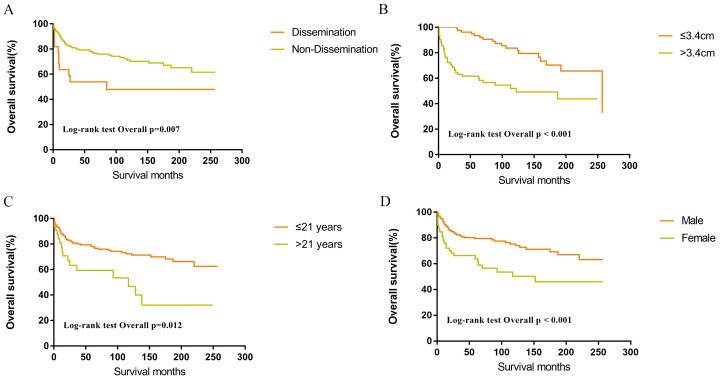
Survival curves of overall survival for dissemination **(A)**, tumor size **(B)**, age **(C)**, sex **(D)** in patients with NGGCTs.

**Figure 4:**
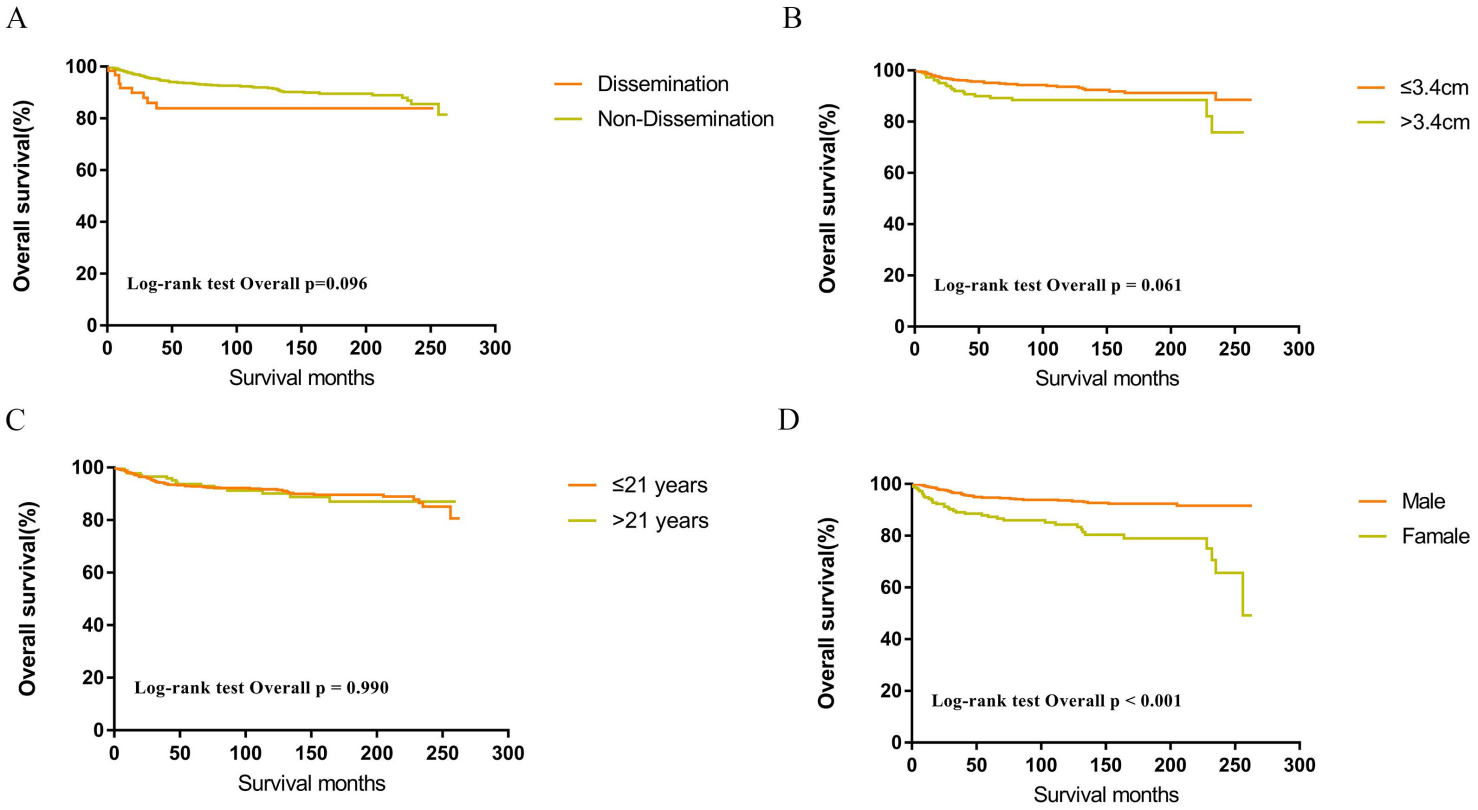
Survival curves of overall survival for dissemination **(A)**, tumor size **(B)**, age **(C)**, sex **(D)** in patients with germinomas.

Within the SEER database, CNS GCTs patients underwent varied treatment regimens, as illustrated in **Table 2**. The survival analysis of patients with germinomas and NGGCTs undergoing differing treatment regimens is illustrated in **Figures 2A, C**. Notably, among germinoma patients, those undergoing only surgical intervention displayed significantly worse prognostic outcomes compared to alternative treatments (P<0.001). We further investigated prognostic differences between patients opting for single treatment modes (radiotherapy, chemotherapy, or surgery) versus those choosing combined modality therapy (radiotherapy plus chemotherapy, radiotherapy plus surgery, chemotherapy plus surgery, or trimodal therapy combining chemoradiotherapy with surgery), visually depicted in **Figures 2B, D**. Additionally, in patients with NGGCTs, age over 21, tumor size larger than 3.4 cm, tumor dissemination, and female gender are associated with an adverse prognosis (**Figure 3**). Conversely, in those with germinomas, only female patients demonstrate a less favorable prognosis, as indicated in **Figure 4**.

### 3.3 Correlations among variables

**Figure 5:**
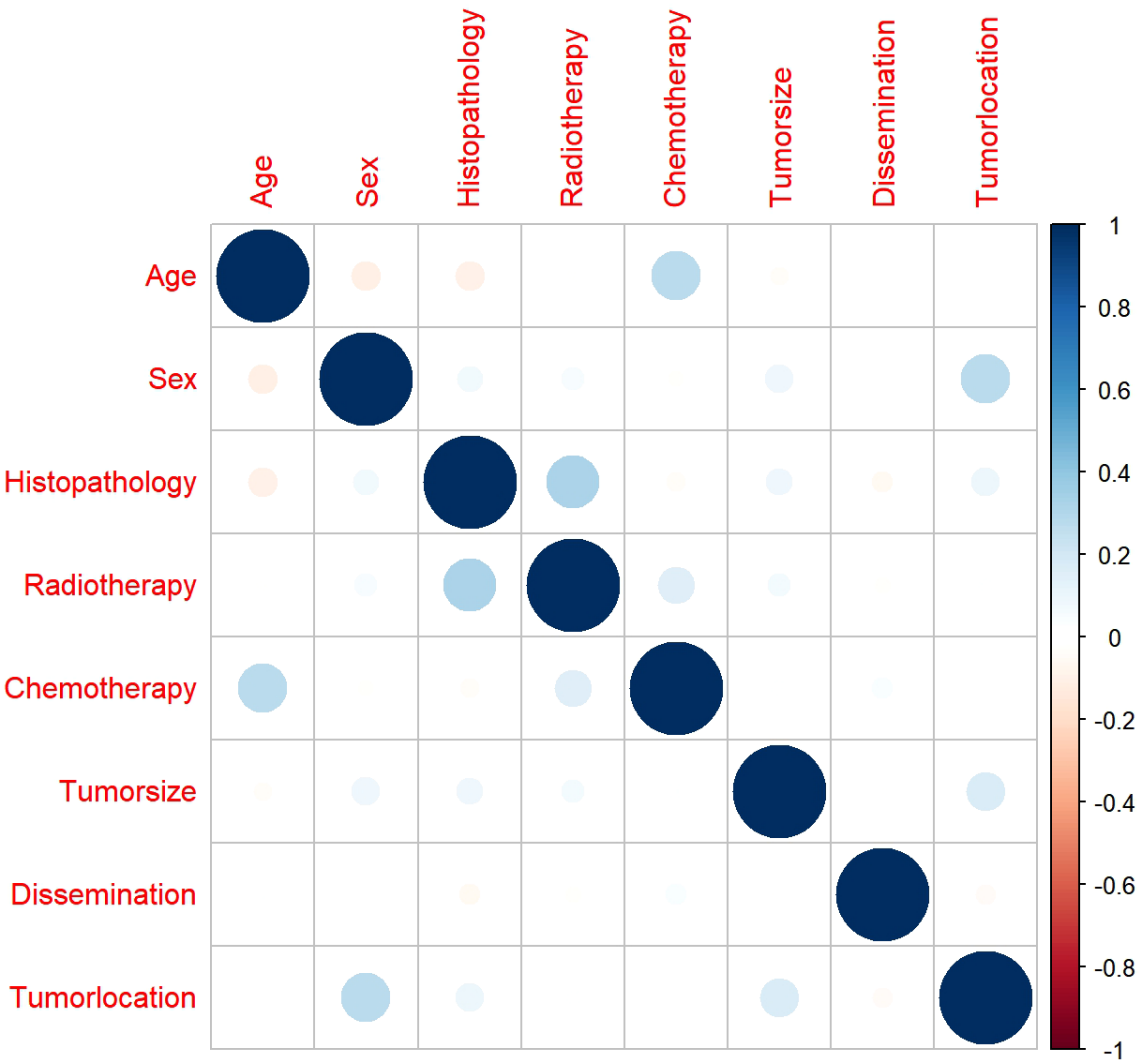
The results of correlation analysis between all included variables.

Before we performed the Cox regression analysis, Spearman’s correlation analysis was used to ensure that there was no collinearity existed between screened variables. The results demonstrated that all pairwise correlation coefficients were below 0.5, indicating only weak correlations between variables and confirming the absence of significant collinearity (**Figure 5**).

### 3.4 Univariate and multivariate Cox regression analysis in the development cohort

**Figure 6:**
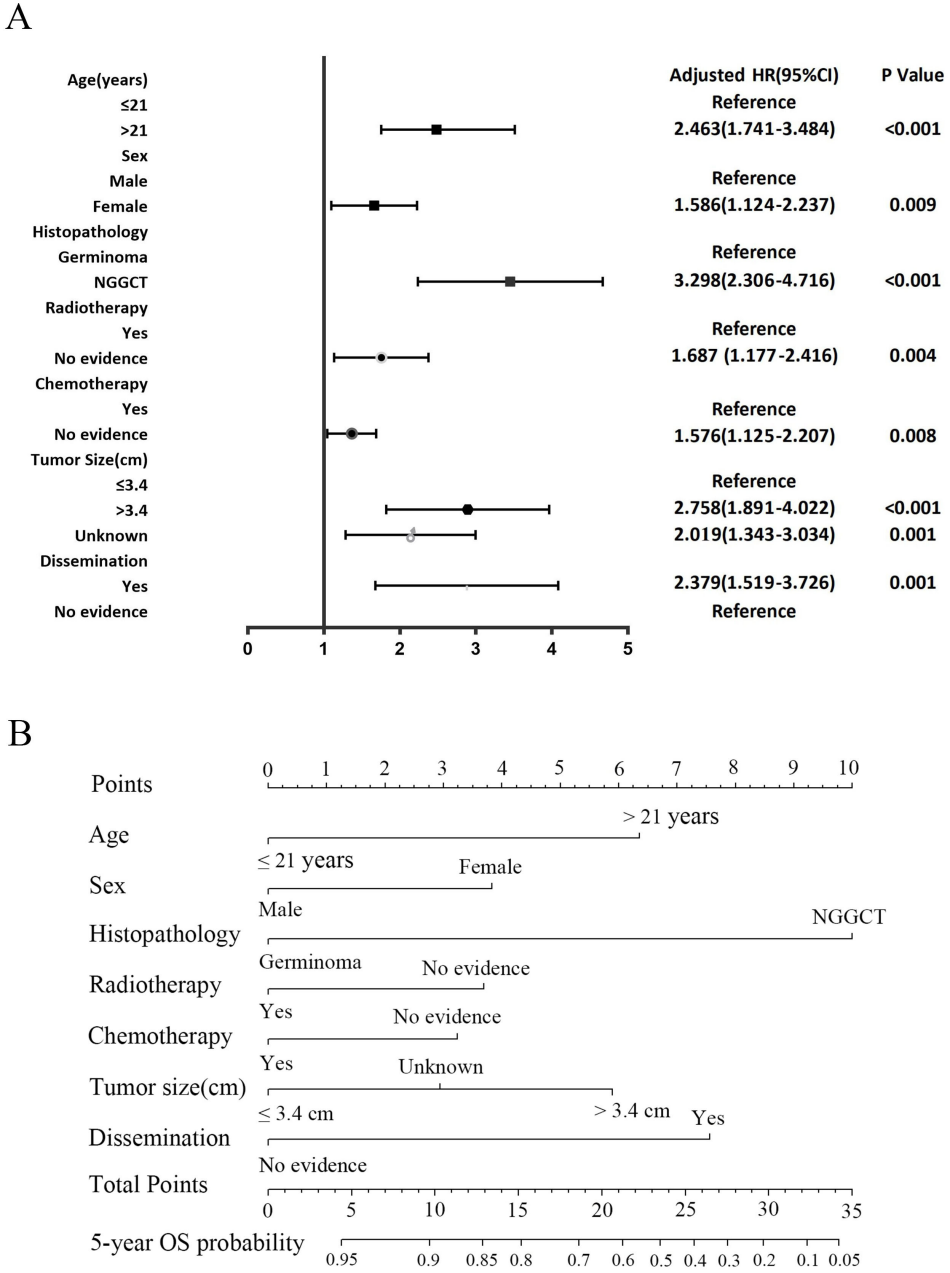
The forest plot of Cox proportional hazard ratio (HR) and 95% confidence intervals (CI) for overall survival of CNS GCTs patients **(A)**. Nomogram predicting the 5-year overall survival rate for CNS GCTs patients **(B)**.

In the development cohort, a range of prognostic factors was assessed, comprising sex, age, race, histopathology, tumor dissemination, location, tumor size, radiotherapy, chemotherapy and surgery. Through univariate Cox regression analysis, all these variables, except for race and surgery, were identified as potential prognostic factors. Subsequently, the multivariate Cox regression analysis identified seven factors as independent prognostic factors for OS: age, sex, histopathology, dissemination, tumor size, radiotherapy, and chemotherapy (**Table 4**). A forest plot was utilized to depict the impact and level of contribution of each independent prognostic factor in relation to the hazard ratio (HR) (**Figure 6A**).

**Table 4:** Univariate and multivariate cox regression analysis for overall survival in CNS GCTs patients in the development cohort.

Characteristics	Univariate analysis		Multivariate analysis	
	HR (95% CI)	P	HR (95% CI)	P
Age (years)				
<=21	Reference		Reference	
>21	1.944(1.408-2.684)	<0.001	2.463(1.741-3.484)	<0.001
Sex				
Male	Reference		Reference	
Female	1.751(1.258-2.436)	0.001	1.586(1.124-2.237)	0.009
Race				
White	Reference		–	–
Black	0.749(0.393-1.427)	0.379	–	–
Others	0.879(0.590-1.310)	0.527	–	–
Tumor location				
Pineal gland/sellar region	Reference		Reference	
Other sites	1.996(1.465-2.719)	<0.001	1.305(0.939-1.814)	0.113
Histopathology				
Germinomas	Reference		Reference	
NGGCTs	4.074(2.999-5.535)	<0.001	3.298(2.306-4.716)	<0.001
RT				
Yes	Reference		Reference	
No evidence	2.848(2.084-3.893)	<0.001	1.687 (1.177-2.416)	0.004
CT				
Yes	Reference		Reference	
No evidence	1.733(1.274-2.358)	<0.001	1.576(1.125-2.207)	0.008
Surgery				
Yes	Reference		–	–
No	0.889(0.655-1.206)	0.448	–	–
Tumor Size (cm)				
<=3.4	Reference		Reference	
>3.4	3.205(2.237-4.592)	<0.001	2.758(1.891-4.022)	<0.001
Unknown	2.156(1.444-3.220)	<0.001	2.019(1.343-3.034)	0.001
Tumor dissemination				
No evidence	Reference		Reference	
Yes	2.265(1.457-3.519)	<0.001	2.379(1.519-3.726)	<0.001

p < 0.05 was considered significant.

NGGCT, non-germinomastous germ cell tumor; RT, Radiotherapy; CT, Chemotherapy; HR, Hazard ratio.

### 3.5 Construction and evaluation of nomogram

**Figure 7:**
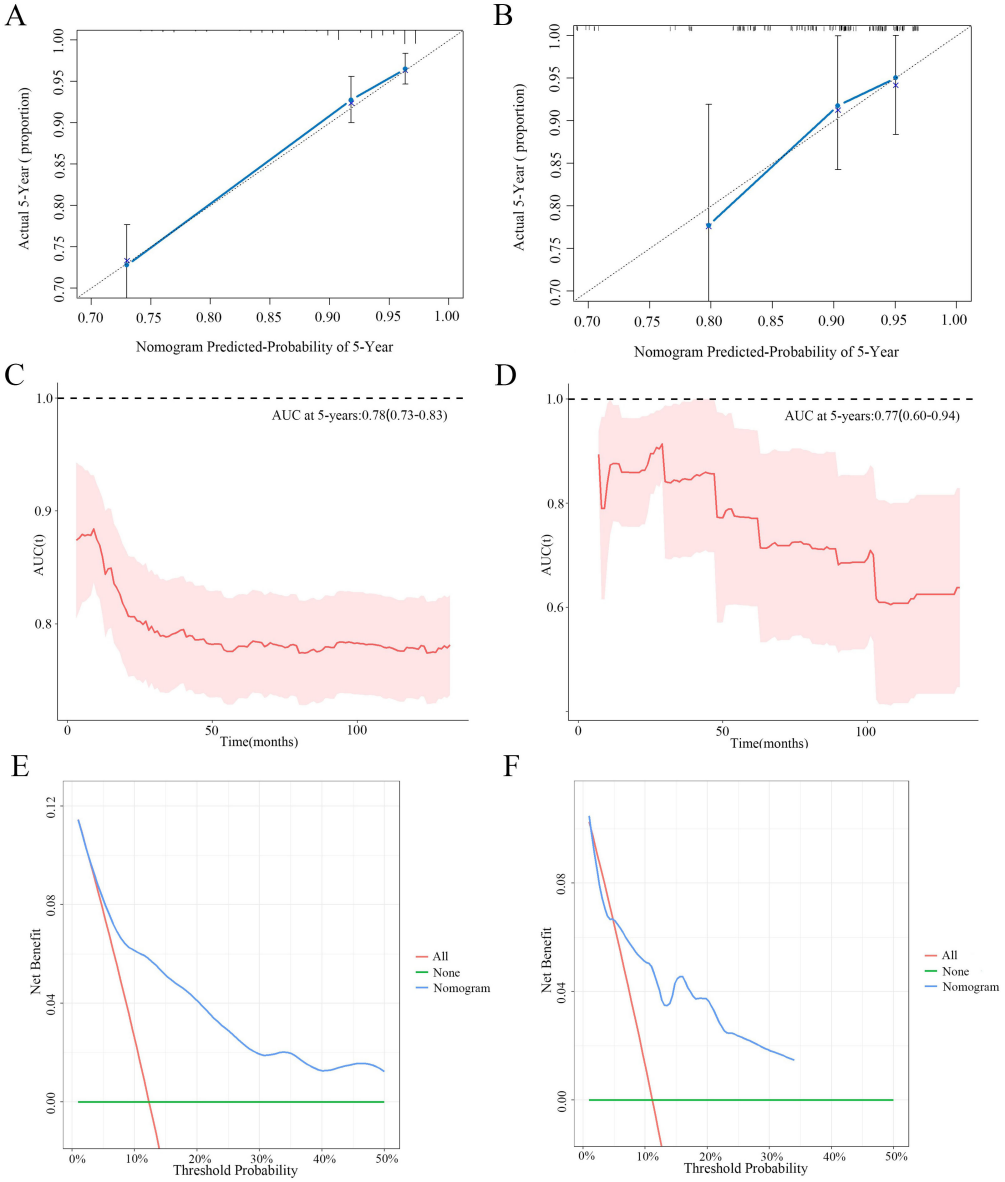
The calibration curves for predictions of 5-year overall survival rate in the development cohort **(A)** and validation cohort **(B)**. Time-dependent AUC of using the nomogram to predict overall survival probability in the development cohort **(C)** and validation cohort **(D)**. Prognostic decision curve analysis (DCA) of CNS GCTs patients within 5-year survival in the development cohort **(E)** and validation cohort **(F)**.

The nomogram for the 5-year OS rate was developed by integrating all prognostic factors identified through multivariate Cox regression analysis (**Figure 6B**). The nomogram was validated through bootstrap analyses with 1000 resamples. The internal and external validation cohorts exhibited favorable discrimination of the nomogram, with C-index values of 0.773 (95% CI, 0.734 - 0.812) and 0.712 (95% CI, 0.599-0.825), respectively. The calibration curves illustrated a high agreement between the observed 5-year OS probability in the development and validation cohorts and the predicted OS from the nomogram (**Figures 7A, B**). Furthermore, the time-dependent area under the curve (AUC) was > 0.7 for predicting 5-year OS in both the development and validation cohorts (**Figures 7C, D**). The 5-year DCA further supported the reliability of our nomogram as a predictor of survival in patients with CNS GCTs (**Figures 7E, F**). These results offer strong evidence supporting the practical application of the nomogram.

### 3.6 Nomogroups of risk grouping system

**Figure 8:**
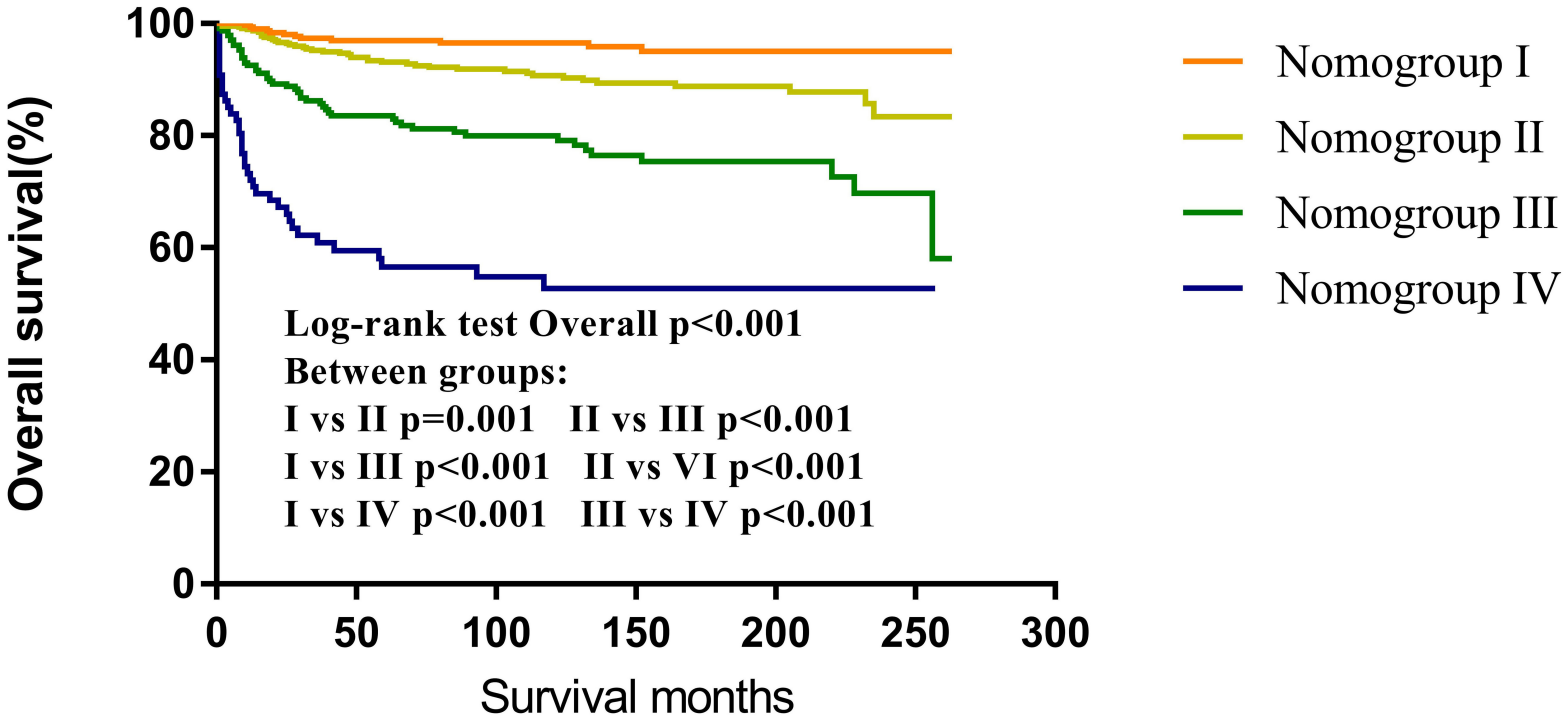
Survival curves of overall survival according to Nomogroups.

According to the nomogram, CNS GCTs patients from the SEER cohort were classified into four prognostic groups based on their total point scores utilizing X-tile software. The risk nomogroups were as follows: nomogroup I (0–3 points), nomogroup II (4–11 points), nomogroup III (13–19 points), and nomogroup IV (≥20 points). Notably, Kaplan-Meier analysis revealed statistically significant differences in prognosis among the four risk nomogroups (**Figure 8**).

## 4 Discussion

In this study, given that most patients with CNS GCTs survive for over 5 years, the primary endpoint of the nomogram was chosen as the 5-year OS rate. the primary endpoint of the nomogram was chosen as the 5-year OS rate. It has been reported that germinoma patients can achieve a 5-year OS rate exceeding 90% due to their high sensitivity to radiotherapy and chemotherapy (16–18). In contrast, NGGCTs often show reduced response to radiotherapy and chemotherapy, resulting in a 5-year OS rate ranging from 60% to 70% after receiving chemoradiotherapy (19, 20). Consistent with published data, our analysis of the SEER cohort unveiled a 5-year OS rate of 93.0% for patients with germinomas, whereas the 5-year OS rate for NGGCTs stands at 73.3%. Thus, the prognosis of CNS GCT patients is primarily influenced by the histopathological type. Challenges related to surgery or patients’ reluctance for biopsy hinder the use of pathological diagnosis in clinical settings. Hence, clinical diagnosis is performed for patients who do not have pathological specimens available. Patients in the SYSUCC cohort without accessible pathological specimens had their clinical diagnosis determined in accordance with the consensus guidelines for CNS GCTs management (14). Our analysis revealed similar 5-year OS rates in clinically diagnosed and pathologically confirmed instances of germinomas and NGGCTs. These findings support the reliability of clinical diagnosis in this study, justifying the inclusion of clinically diagnosed patients in the validation cohort. This inclusion not only augmented the sample size but also improved the predictive precision of our assessment model.

It is generally believed that the outcome of CNS GCTs is not associated with tumor size, while several studies have suggested that a larger tumor size is a poorer prognostic factor for CNS GCTs (21–24). In the current study, multivariate Cox regression revealed that a tumor diameter greater than 3.4 cm was an independent risk factor for overall survival. Interestingly, subgroup analysis found that the negative impact of a large tumor size on prognosis was observed only in NGGCTs and not in germinomas. This variation in the effect of tumor size on prognosis could be due to the heightened sensitivity of germinomas to chemoradiotherapy. Consequently, the influence of tumor size on prognosis might be confined to patients with NGGCTs, showing no discernible impact on patients with germinomas.

Chen et al. reported that survival rates were comparable between patients with disseminated germinomas and those without dissemination (25). Tumor dissemination in this study served as a significant independent prognostic factor for patients with CNS GCTs in the SEER cohort. Notably, subsequent subgroup analysis revealed no statistically significant variance in survival rates between patients with disseminated germinomas and those without dissemination. Furthermore, for CNS GCTs patients with dissemination, germinomas exhibited a more favorable prognosis than NGGCTs. These findings can be attributed to the higher radiosensitivity of germinomas.

Radiotherapy plays a vital role in CNS GCTs management. However, considering the neurological side effects linked to radiotherapy, the current strategy emphasizes shrinking tumor size using chemotherapy prior to commencing radiation therapy (16, 18, 26). Analysis within the SEER cohort revealed that CNS GCTs patients receiving chemoradiotherapy and radiotherapy experienced extended survival compared to those treated solely with chemotherapy. Typically, patients receiving chemotherapy alone are thought to encounter elevated recurrence rates and inferior prognoses compared to those receiving radiotherapy (27, 28). Nevertheless, the Cox proportional hazards model analysis conducted in this study revealed that the prognosis does not vary among patients with germinomas undergoing radiotherapy alone and chemotherapy alone, this observation could be attributed to certain patients who underwent chemotherapy alone also receiving extra treatments, like radiotherapy, not recorded in the SEER database. Furthermore, our discovery indicated that standalone surgery yielded the poorest prognosis for germinoma, emphasizing the limitation of relying solely on surgical intervention, aligning with present recommendations (29).

NGGCTs exhibit inherent resistance to radiation therapy, resulting in the infrequency of using radiotherapy as a monotherapy in current clinical practices (1, 16, 30). Present therapeutic strategies for NGGCTs emphasize the multidisciplinary approach of integrating radiotherapy with chemotherapy (5, 31). Controversy exists regarding the effectiveness of incorporating neurosurgical procedures into the core of chemoradiotherapy for CNS GCTs, mainly because of the notable responses of the majority of histological subtypes of CNS GCTs to radiotherapy and chemotherapy. Aoyama et al. studied 33 pediatric cases of CNS GCTs, illustrating that surgical excision, in conjunction with combined chemotherapy and limited radiotherapy, resulted in a 5-year overall survival rate surpassing 69% (32). In this research, no distinction in prognosis was found between the combined treatment of surgery with chemoradiotherapy and chemoradiotherapy, suggesting that the advantage of adding surgery to chemoradiotherapy is not conclusively proven. Presently, surgical intervention is deemed suitable for suspected teratomas, residual lesions after chemoradiotherapy, and for alleviating life-threatening space-occupying manifestations (14, 33). However, due to the limited sample sizes of NGGCTs in this study, further prospective clinical trials must be done to assess the efficacy of combined surgery and chemoradiotherapy treatment for NGGCTs. Furthermore, our findings indicate that combination therapy offers substantial benefits in enhancing the survival outcomes of CNS GCTs patients, whether germinomas or NGGCTs, which indicated that future therapeutic approaches for CNS GCTs favor a multidisciplinary integrated therapy.

Gender variations are evident in CNS GCTs, with a higher prevalence among male patients compared to female patients (29), aligning with our research findings. Additionally, Acharya et al. found an increased mortality risk in female germinoma patients compared to their male counterparts (34). Nevertheless, a study by Steven et al. revealed comparable prognoses among male and female germinoma patients (35). Remarkably, our study detected survival differences based on gender in CNS GCTs. Although the underlying reason remains unclear, we propose that the gender disparities in CNS GCTs incidence and outcomes stem from complex interactions encompassing DNA methylation discrepancies, epigenetic alterations, and brain sexual differentiation influenced by exposure to sex hormones (36). Accordingly, additional prospective investigations are imperative to explore the influence of gender on prognosis.

Emphasizing the strengths of our nomogram is crucial. Firstly, our nomogram demonstrates extensive generalizability by integrating databases from both Western and Eastern populations, rendering it suitable for diverse regions. Secondly, most research on CNS GCTs faces limitations with small sample sizes. In contrast, our dataset is sourced from the SEER database, encompassing 18 cancer registries and reflecting around 34.6% of the US population. This extensive dataset greatly enhances the reliability and accuracy of our research. Lastly, by utilizing the risk grouping system, clinicians can also identify different risk stratification of CNS GCTs patients, which assists decision making about the risk and benefit of treatment or care options.

However, being a retrospective study, our study has limitations. Firstly, the recently updated SEER database categorizes “No/Unknown” as a single group, with regards to chemotherapy and radiotherapy. Secondly, detailed data on chemotherapy and radiotherapy therapy such as chemotherapy regimen, radiotherapy dose and radiotherapy methods were not accessible in the SEER database. Thirdly, information about several important clinicopathological parameters such as tumor size was not complete. In order to mitigate potential biases, we retained this data despite its classification as “unknown”. Finally, limited by the constraints on case numbers of the SEER database, the NGGCTs group had only one patient who received radiotherapy exclusively, underscoring the need for extensive studies to explore the impact of radiotherapy on NGGCTs prognosis with larger sample sizes.

## 5 Conclusion

In conclusion, we have successfully identified prognostic factors for CNS GCTs and developed a nomogram that integrates seven clinicopathological and treatment-related variables. This nomogram accurately predicts the prognosis of patients with CNS GCTs and provides clinicians with personalized treatment options and guidance for long-term management.

## Data Availability

The raw data supporting the conclusions of this article will be made available by the authors, without undue reservation.

## Supplementary material

Supplementary Figure 1 Survival curves with the log-rank tests of overall survival according to histological subtype.

Supplementary Figure 2 Survival curves with the log-rank tests of overall survival according to treatment regimen. RT, radiotherapy; CT, chemotherapy.

Supplementary Figure 3 Survival curves with the log-rank tests of overall survival in patients with disseminated according to histological subtype.
