# Supplementary material for: Development and validation of a nomogram for predicting overall survival in patients with primary central nervous system germ cell tumors
Source: Front Immunol. 2025 Aug 20;16:1630061. doi: 10.3389/fimmu.2025.1630061 (PMC12404971; doi:10.3389/fimmu.2025.1630061)
Supplement: Supplementary file 1 [file Table1.docx]

**Supplementary Table1.** Tumor marker for clinically diagnosed patients in the SYSUCC cohort

| Variables | Total patients (n=66) | | Germinoas (n=42) | | NGGCTs (n=24) | |
| --- | --- | --- | --- | --- | --- | --- |
|  | **No.** | **%** | **No.** | **%** | **No.** | **%** |
| HCG (IU/L) |  | | | | | |
| ≤ 50 | 46 | 69.7 | 42 | 100 | 4 | 16.7 |
| > 50 | 20 | 30.3 | 0 | 0 | 20 | 83.3 |
| AFP (ng/mL) |  | | | | | |
| ≤ 25 | 58 | 87.9 | 42 | 100 | 16 | 66.7 |
| > 25 | 8 | 12.1 | 0 | 0 | 8 | 33.3 |

**Abbreviations:** HCG, human chorionic gonadotropin; AFP, alpha-fetoprotein; NGGCT, non-germinomatous germ cell tumor.
