# Supplementary figures and images for: Development and validation of a nomogram for predicting overall survival in patients with primary central nervous system germ cell tumors
Source: Front Immunol. 2025 Aug 20;16:1630061. doi: 10.3389/fimmu.2025.1630061 (PMC12404971; doi:10.3389/fimmu.2025.1630061)

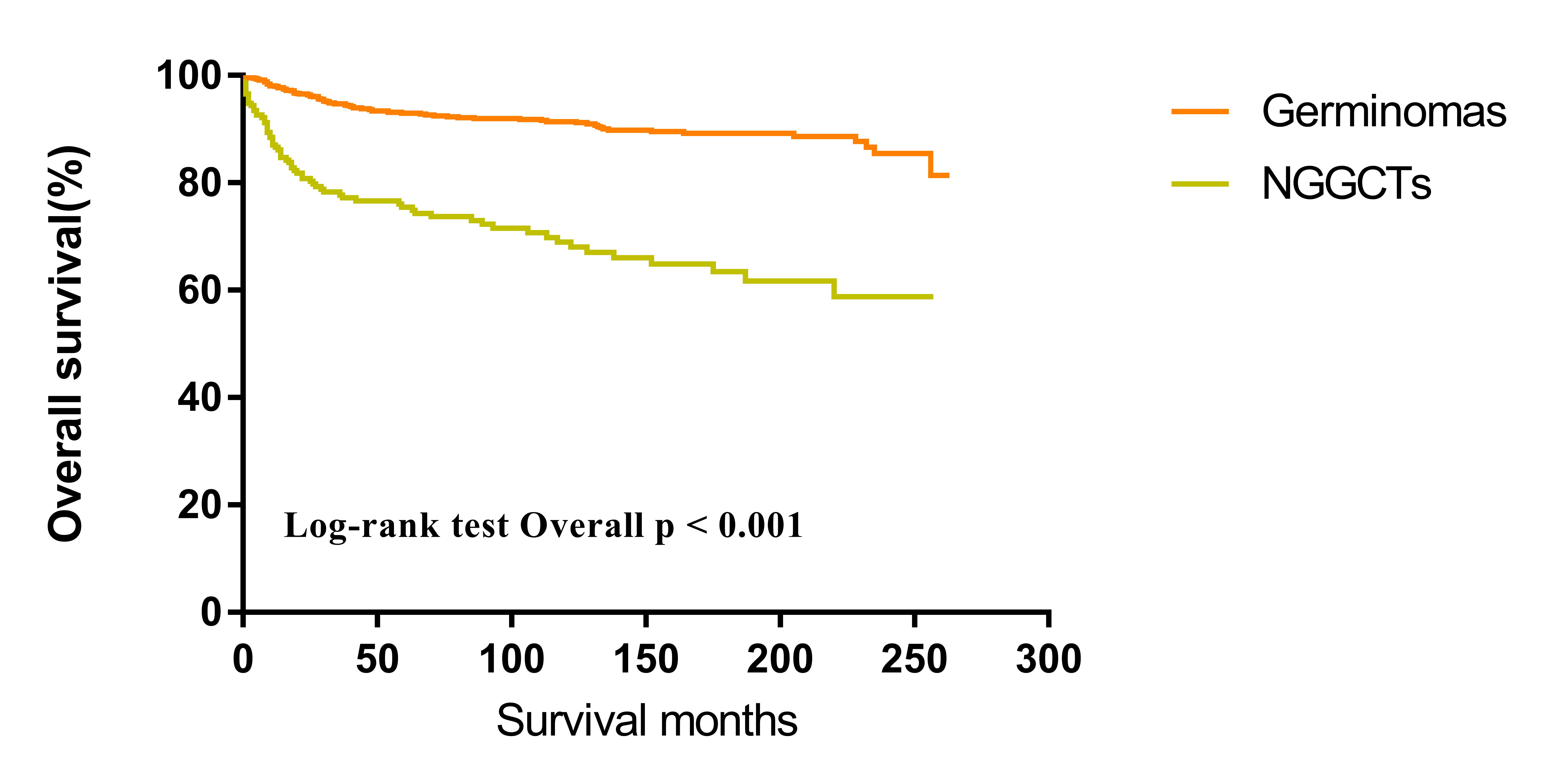

Supplement: Supplementary Figure 1 — Survival curves with the log-rank tests of overall survival according to histological subtype. [file Image1.jpeg]

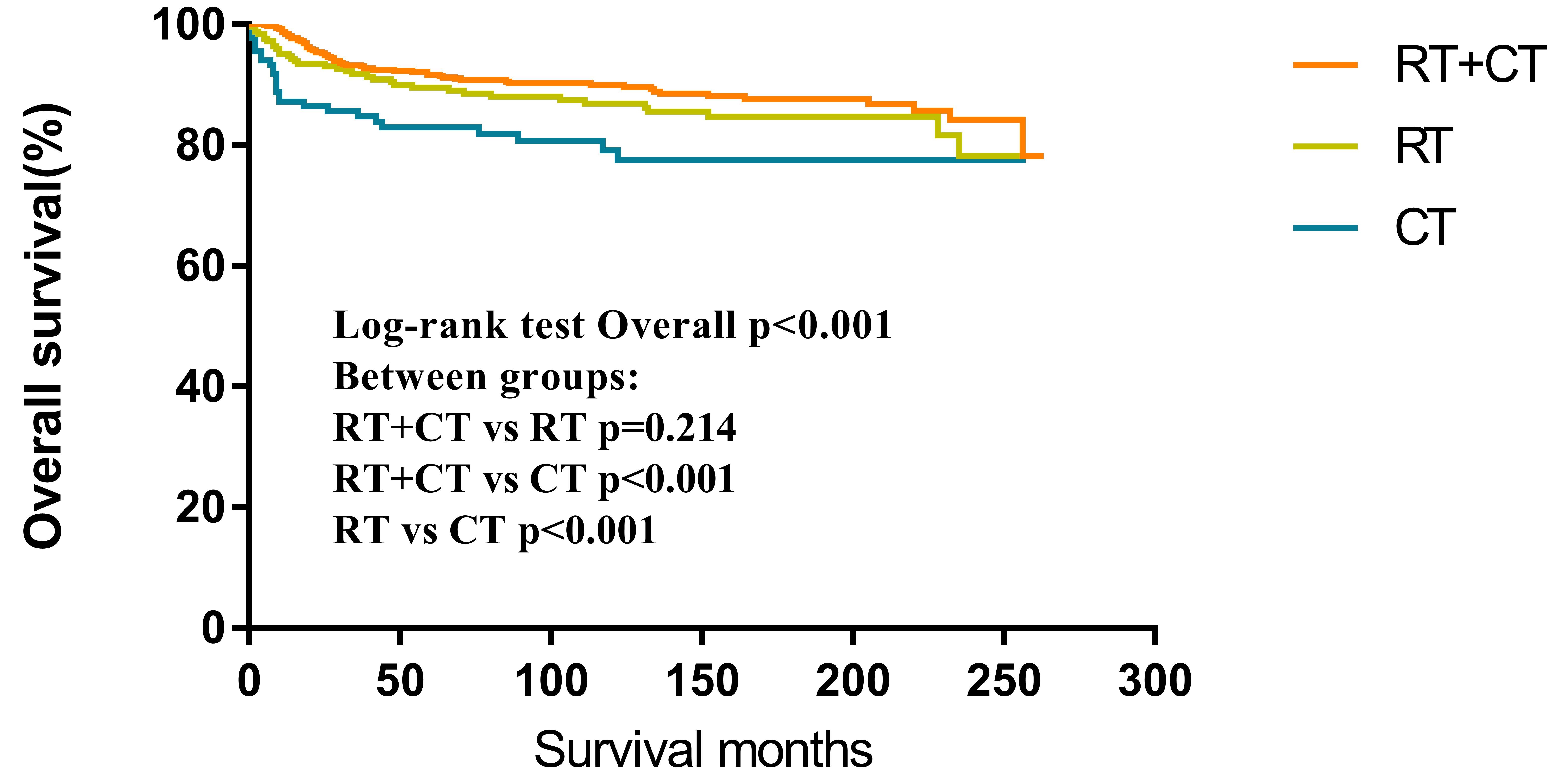

Supplement: Supplementary Figure 2 — Survival curves with the log-rank tests of overall survival according to treatment regimen. RT, radiotherapy; CT, chemotherapy. [file Image2.jpeg]

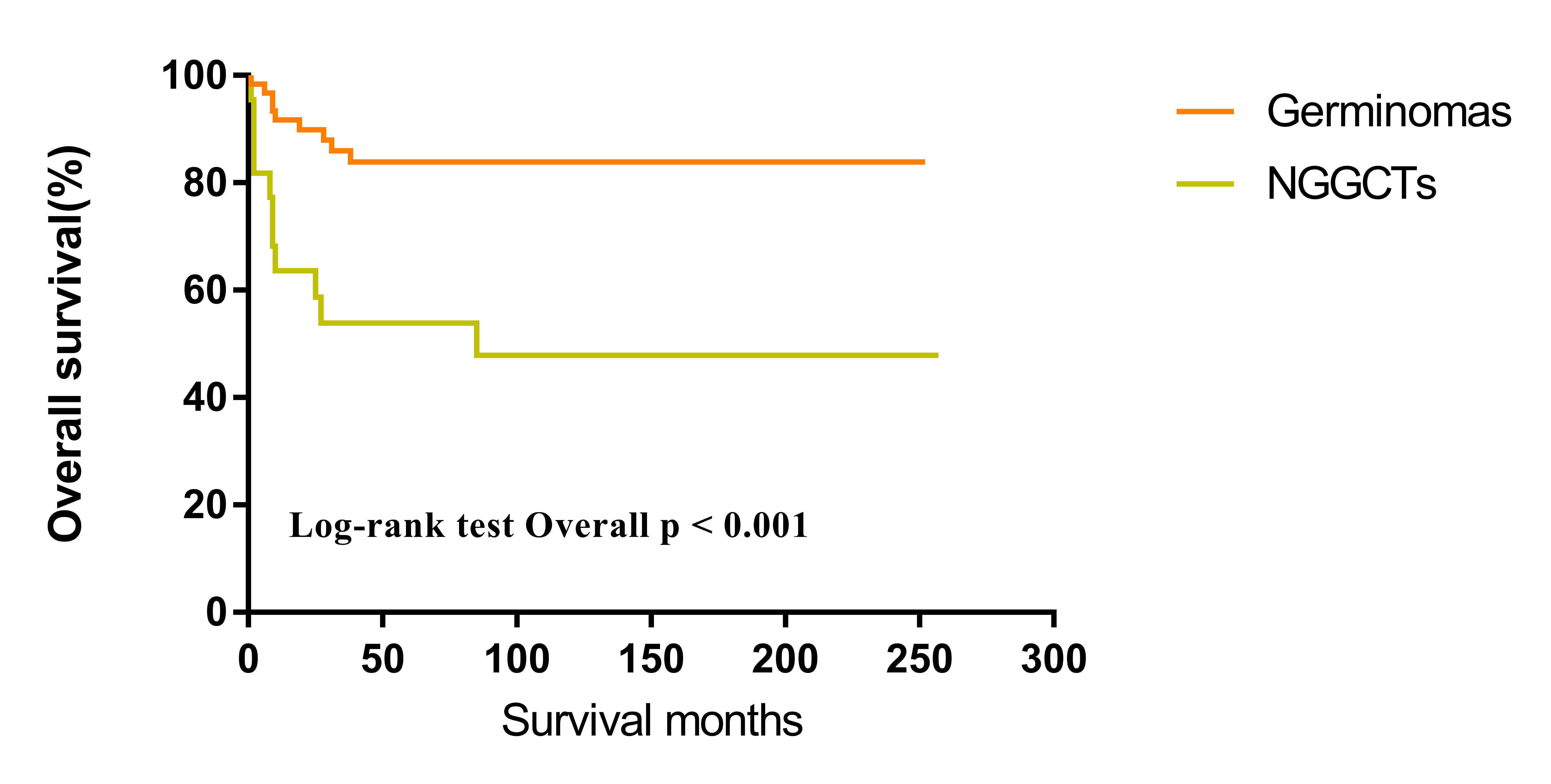

Supplement: Supplementary Figure 3 — Survival curves with the log-rank tests of overall survival in patients with disseminated according to histological subtype. [file Image3.jpeg]
